# Identification of IL6 as a susceptibility gene for major depressive disorder

**DOI:** 10.1038/srep31264

**Published:** 2016-08-09

**Authors:** Chen Zhang, Zhiguo Wu, Guoqing Zhao, Fan Wang, Yiru Fang

**Affiliations:** 1 Division of Mood Disorders, Shanghai Mental Health Center, Shanghai Jiao Tong University School of Medicine, Shanghai, China

## Abstract

Our previous work implied that interleukin 6 (IL6) may be a biological marker for major depressive disorder (MDD). In this study, we performed a comprehensive genetic study to determine the association between the gene encoding IL6 (*IL6*) and MDD in Han Chinese. There were 50 drug-naïve MDD patients and 50 healthy controls undergoing an mRNA expression study. A sample of 772 patients with MDD and 759 healthy controls were used for genetic analysis. Next, we performed an eQTL analysis to identify whether risk SNP(s) is associated with *IL6* expression in brain. Our results showed that patients with MDD have higher levels of *IL6* than healthy controls (*P* = 0.008). The SNP rs1800797 has a significant association with MDD (*P* = 0.01) in a dominant model. The eQTL analysis showed a marginally significant association between the rs1800797 and *IL6* expression in the frontal cortex (*P* = 0.087). Our preliminary findings are suggestive of an association between rs1800797 and the risk of MDD. Further investigations are required to evaluate this association in larger samples to increase statistical power, and to examine the correlation between rs1800797 and *IL6* methylation patterns.

Major depressive disorder (MDD) is a severe mental disorder, typically characterized by a cluster of emotional and somatic symptoms at psychological level^1^, and abnormal brain connectivity and functioning at physical level^2^. MDD is prevalent in excess of 17% among Han Chinese^3^ and also becoming a leading cause of disability and mortality in the worldwide. Thus, it is one of the most urgent challenges for current psychiatric research to understand the pathogenesis of MDD

It has been confirmed that the heritability of MDD is estimated up to 70%^4^ and recent studies into its genetic etiology have detected a number of susceptibility genes and chromosomal regions implicated with immune system^5^,^6^. Clinical studies have widely demonstrated aberrant inflammation profiles of MDD patients in either central neural system (CNS) or peripheral tissues^7^,^8^. Therefore, one emerging hypothesis for this association is that chronic low-grade activation of inflammation and the immune system likely contribute to some of the biological mechanisms in the development of MDD^9^.

Our previous work using whole-genome cRNA microarrays found that genes associated with MDD were enriched in interleukin 6 (IL6)-mediated signaling events^10^. Similar results were also reported in the Netherlands^11^. IL6 is a multifunctional cytokine that regulates the growth and differentiation of various tissues, and plays an important role in the immune response and acute phase reactions^12^. Goldsmith *et al*.^13^ performed a meta-analysis of blood cytokines in 18 studies for MDD. The authors observed enhanced level of IL6 in acutely MDD patients, and following treatment for acute phase, IL6 level significantly decreased in patients with MDD. In CNS, IL6 acts as a neurotrophic cytokine expressed in both neurons and glia^14^, whose level is also reported to be increased in the cerebrospinal fluid of MDD patients^15^. Such findings provided suggestive evidence for the role of IL6 in the pathophysiology of MDD.

At the molecular level, the human gene encoding IL6 (*IL6*) is mapped to chromosome 7p15, a candidate region previously implicated in MDD^16^. Unsurprisingly, genetic variations of *IL6* have been reported to modulate the chronic stress exposure in the development of depressive symptoms^17^, and increase the risk of interferon-induced depression^18^. Based on this premise, it is plausible that *IL6* is likely to be a promising candidate gene for MDD susceptibility.

In this study, we hypothesized that *IL6* may be a susceptibility gene for MDD. First, we analyzed the *IL6* mRNA expression difference between drug-naïve MDD patients and normal controls. Given the relevance of genetic variations of *IL6* to depressive symptoms, we subsequently investigated whether *IL6* is genetically associated with MDD among a Chinese Han population. As a third aim, we performed an eQTL (expression quantitative trait loci) analysis via an available database to investigate the potential role of the risk SNP in *IL6* mRNA expression in brain.

## Results

**Figure 1:**
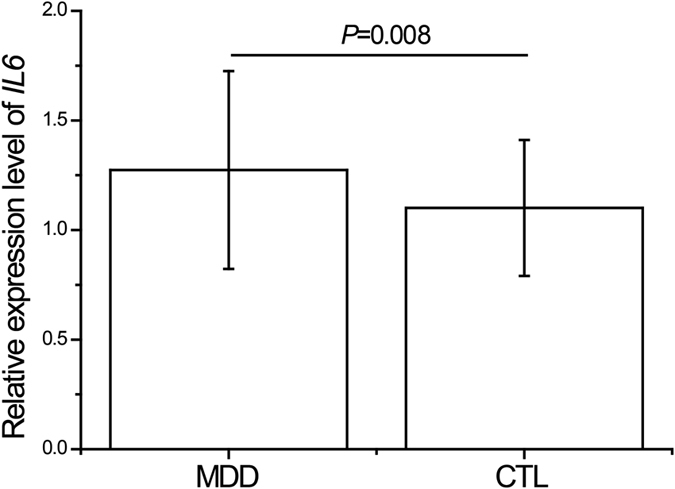
Expression levels of *IL6* mRNA in peripheral blood in drug-naïve patients with major depressive disorder and healthy controls. *IL6* mRNA was normalized to that of *GAPDH*. MDD, major depressive disorder patients (n = 50); CTL, control controls (n = 50).

In total, 50 drug-naïve patients with MDD and 50 healthy controls were measured for peripheral *IL6* mRNA expression. The two cohorts were well matched in terms of age, gender and smoking status, but significant difference was observed in body mass index (BMI) (*P* = 0.03, Supplementary Table S1). Figure 1 showed that patients with MDD have higher levels of *IL6* than healthy controls (*P* = 0.008).

Genotype distributions of all studied polymorphisms in control group were consistent with the Hardy-Weinberg equilibrium (HWE) (*P* > 0.05). Statistical analyses of the data for the five polymorphisms were presented in Table 1. The SNP rs1800797 showed a significant association with MDD (*P* = 0.01) in a dominant model. The frequency of the A allele of rs1800797 was significantly higher among MDD patients than that among the controls (OR = 4.72, 95% CI: 1.60–13.89, *P* = 0.01). The pairwise LD among the 5 SNPs is presented in Supplementary Figure S1. Strong LD was observed between rs1524107 and rs2069837 (*D*’ = 1.00, *r*^2^ = 0.57). Supplementary Table S2 listed all *P* values corresponding to the haplotypes, with a haplotype frequency less than 3% being dropping. We did not find any significant association of the haplotypes consisting of rs1524107 and rs2069837 with MDD.

**Figure 2:**
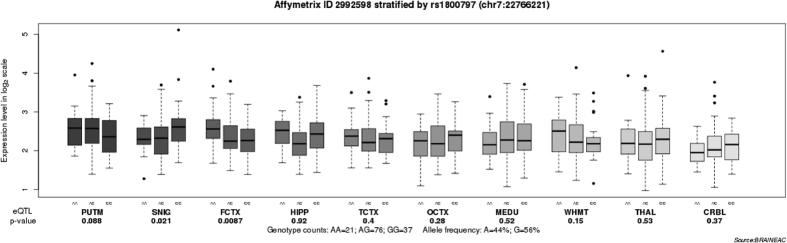
Association of rs1800797 with *IL6* mRNA expression levels in ten brain regions (Affymetrix ID 2992598). Data were extracted from the BRAINEAC database (http://caprica.genetics.kcl.ac.uk/BRAINEAC/). SNIG, substantia nigra; PUTM, putamen (at the level of the anterior commissure); MEDU, the inferior olivary nucleus (sub-dissected from the medulla); THAL, thalamus (at the level of the lateral geniculate nucleus); OCTX, occipital cortex; HIPP, hippocampus; FCTX, frontal cortex; TCTX, temporal cortex; WHMT, intralobular white matter; CRBL, cerebellar cortex.

We then performed an eQTL analysis to investigate whether SNP rs1800797 influences the *IL6* expression in the brain. As shown in Fig. 2, we observed a marginally significant association between the rs1800797 and *IL6* expression in the frontal cortex (*P* = 0.087). Data showed that carriers with A allele have significantly higher levels of *IL6* expression in frontal cortex that those without A allele.

## Discussion

As MDD etiology is known to be linked to inflammation in at least some cases and immune response has been proven to be genetically influenced^19^, it is speculated that genetic factors in immune dysfunction may be involved in the pathophysiology of MDD^20^. IL6, a key proinflammatory cytokine^21^, has been reported in the development of MDD in prior literature. Our previous work has provided suggestive evidence for its role in the etiology of MDD in Han Chinese. Herein, we performed a comprehensive analysis to investigate the association of *IL6* with MDD in Chinese Han population. To the best of our knowledge, there is the first study to address this association.

In the first step, we tested the levels of *IL6* mRNA expression in drug-naïve patients with MDD and healthy controls. Our results showed a significant higher level of *IL6* expression in MDD group than that in control one. This is in line with previous reports^22^,^23^. On the other hand, the elevated level of *IL6* in MDD patients can be reduced to a normal level after antidepressant treatment as the depressive symptoms improved^23^,^24^. Taken together, the above evidence implies that increased *IL6* expression is possibly involved in the etiology of MDD.

Earlier studies have detected the genetic mechanism of *IL6* in depressive symptoms. SNP rs1800795, a functional polymorphism in the promoter of *IL6*, was reported to increase the risk of stress-^17^ and interferon treatment-^18^ induced depression. However, it is not known about the role of *IL6* variations in the etiology of MDD. Thus, we carried out a genetic study to evaluate the association of *IL6* with MDD in Han Chinese. We found a MDD-associated SNP rs1800797 in a dominant model, and the frequency of the A allele of rs1800797 was significantly higher among MDD patients than that among the controls. The frequency of A allele in controls (0.5%) is similar to the reported data in Han Chinese (0.3%)^25^. Prior literature reported that rs1800797 polymorphism is associated with a number of human diseases, such as asthma^26^, type II diabetes^27^ and leprosy^28^ that were reported to have a high morbidity prevalence with MDD^29^,^30^,^31^. SNP rs1800797 presents in the promoter region of *IL6*. Variations in this region may lead to functional alteration of *IL6* and ultimately influence the occurrence of human diseases^32^. Our eQTL analysis showed that the rs1800797 polymorphism have a regulatory effect on *IL6* expression in the frontal cortex and individuals with A allele of rs1800797 have higher **levels** of *IL6* expression than those without the A allele. These results imply that elevated levels of *IL6* expression in the frontal cortex may be a risk factor for the development of MDD.

The neural network is believed to modulate aspects of normal emotional behavior and implicated in the pathophysiology of MDD^33^. Advances in neuroimaging techniques offer the potential to investigate the neural mechanism underlying MDD. Recently, we scanned patients with MDD and healthy controls using magnetic resonance imaging (MRI) to investigate the alterations in the cortical surface of MDD, and our findings suggest that frontal cortical alteration is a vulnerability to MDD during earlier neurodevelopmental process^34^. A functional MRI experiment showed that MDD patients exhibit abnormal long distance connectivity and dysregulation of large-scale neural networks in medial prefrontal cortex^35^. A number of positron emission tomography (PET) studies have repeatedly identified a decrease in metabolic activity in the prefrontal cortex in patients with MDD^36^,^37^,^38^. Hence, abnormalities in prefrontal cortex may be important for investigations of the pathophysiology of MDD. Furthermore, Setiawan *et al*.^7^ applied PET to examine a marker of neuroinflammation, translocator protein (TSPO) binding *in vivo*, in order to determine the neuroinflammatory hypothesis of MDD. Their results showed that the magnitidue of TSPO density elevation was 26% in the prefrontal cortex of patients with MDD than that of controls. This suggests that neuroinflammation activation leading to abnormal function in prefrontal cortex may contribute to the development of MDD. As such, it is remain unknown whether rs1800797 polymorphism is responsible for the MDD-related neuroinflammation in frontal cortex, and this will subsequently need to be investigated in future. Meanwhile, literature indicated that gene expression of *IL6* is regulated by DNA methylation of its promoter region^39^. The region from positions −666 to −426 relative to the transcription state site in *IL6* may be the potential binding sites for methylation^40^. SNP rs1800797 consists of a G to A substitution at the −597 site. We speculated that DNA methylation may explain the underlying mechanism of rs1800797 in the etiology of MDD. This also requires for further clarification.

There are two limitations to the present study. As known that MDD is a mental disorder that originates from brain dysfunction, we measured the peripheral level of *IL6* mRNA expression in this study. Thus, further analyses using brain tissues are needed to validate our results. Second, our results showed that the A allele of rs1800797 was found in 4 out of 759 controls. Although the HWE *P* value for this SNP in controls is 0.94, such a low frequency in a small sample size could potentially bias the HWE and dilute the statistical power. Taking it into consideration, our findings should be considered only preliminary.

In conclusion, we performed a comprehensive analysis to detect the role of *IL6* on the pathophysiology of MDD in Chinese Han population. Our preliminary findings are suggestive of an association between rs1800797 and the risk of MDD. Further investigations are required to evaluate this association in larger samples to increase statistical power, and to examine the correlation between rs1800797 and *IL6* methylation patterns.

## Methods

### Participants

For the expression study, there were 50 drug-naïve MDD patients, and 50 healthy controls recruited from the Division of Mood Disorders, Shanghai Mental Health Center, Shanghai Jiao Tong University School of Medicine. Demographic data on age, gender, smoking status, BMI, alcoholic abuse, duration of illness prior to admission, number of episode, family history of mood disorders was collected. Assessments of the Hamilton Rating Scale for Depression −17 (HRSD-17) were conducted independently by two experienced psychiatrists (interrater reliability, kappa = 0.84)^41^.

For the genetic study, we enrolled the MDD samples from our previous clinical trials: the “OPERATION” (OPtimized trEatment stRAtergies for Treatment-resIstant depressiON) study^42^,^43^ and the “CARE-SSD/MDD” (Construct An Rough Evaluation index system for subsyndromal symptomatic depression and major depressive disorder) study. All patients were diagnosed with MDD strictly according to The Diagnostic and Statistical Manual of Mental Disorders, Fourth Edition (DSM-IV) criteria. Standard diagnostic assessments were supplemented with clinical information obtained by a review of medical records and interviews with family informants. Patients were excluded on the following criteria: (1) those with a lifetime diagnosis of bipolar disorder, schizoaffective disorder, schizophrenia, or another psychotic disorder; as well as (2) female patients who were pregnant, planning to become pregnant, or breast-feeding during the study period.

Control subjects were enlisted from the hospital staff and students of the School of Medicine in Shanghai that were interviewed by a specialized psychiatrist with SCID-P. Subjects with any psychiatric disorder and chronic physical disease were excluded from our analysis^44^,^45^.

All the patients and control subjects were of Han Chinese origin from Shanghai. All procedures were reviewed and approved by Institutional Review Boards of Shanghai Mental Health Center and other participating institutions. This study was performed in accordance with the guidelines laid out in the Declaration of Helsinki as revised in 1989. All subjects were of Han Chinese origin and provided written informed consent before any study-related procedures were performed.

### RNA preparation and Quantitative real-time polymerase chain reaction (qRT-PCR)

On admission, 20 ml peripheral blood of fasting patients and healthy controls were collected between 07:00 am and 09:00 am, to avoid potential diurnal influence. RNA preparation was carried out as previously described^46^.

Relative *IL6* mRNA expression levels were assessed by qRT-PCR with commercially available TaqMan gene expression assays for target gene *IL6* and glyceraldehydes-3-phosphate dehydrogenase (*GAPDH*) as reference gene (Applied Biosystems, CA, USA). All experiments were conducted as referring to our previous studies^45^,^47^,^48^. In each sample, the expression of *IL6* was normalized to the expression of the reference gene *GAPDH*. Results were reported in fold change using 2^−∆∆Ct^.

### SNP selection and Genotyping

We retrieved CHB data from the HapMap database (http://www.hapmap.org) and defined linkage disequilibrium (LD) blocks using Haploview 4.2 (Broad Institute, Cambridge, MA, USA) to set inclusion criteria for tagging SNPs. Haplotype-tagging single nucleotide polymorphisms (htSNPs) with *r*^2^ cutoff > 0.8 and minor allele frequency (MAF) > 0.1 were selected. In total, there are two tag SNPs (rs1524107 and rs2069837) of *IL6* selected for genotyping. Three functional SNPs (rs1800797, −597G/A, rs1800796, −572G/C and rs1800795, −174G/C) within *IL6* were also examined in this study, because the activity of the promoter region of *IL6* is affected by the polymorphisms^49^. Detailed information for these selected SNPs is shown in Supplementary Table S3.

Genomic DNA was isolated from whole blood using a Tiangen DNA isolation kit (Tiangen Biotech, Beijing, China). The five SNPs were detected using multiplex PCR and the SNaPshot assay. The detailed experiment processes were described in our previous publication^50^. All of the sample call rates exceeded 99.7%. Of the collected samples, 10% were repeated for the genotyping assay to ensure quality-control, and the results were 100% concordant.

### Brain eQTL analysis

Converging evidence suggests that MDD originates from abnormal brain functions^51^, and brain samples are presumably appropriate for eQTL analysis of risk SNP(s). Here, we performed an eQTL analysis to identify whether risk SNP(s) is associated with *IL6* expression in brain, using the brain eQTL database (http://caprica.genetics.kcl.ac.uk/BRAINEAC/), a large exon-specific eQTL data set covering ten human brain regions. More detailed information can be found in the original study^52^.

### Statistical analysis

The statistical differences in the characteristics between groups were compared using chi-square test or *t* test. For the expression analysis, ANCOVA was carried out with age, gender, BMI and smoking status as covariates controlled in the model, to minimize the potential effect of these factors on the expression levels of *IL6* mRNA^53^. For the genetic analyses, HWE testing, genotype and allele frequency analyses were conducted using SHEsis (http://analysis.bio-x.cn)^54^. Pairwise linkage disequilibrium of all pairs of SNPs was assessed using Haploview 4.2 (Broad Institute, Cambridge, MA, USA)^55^, and the extent of linkage disequilibrium (LD) was measured by the standardized *D*’ and *r*^2^. Odds ratios (ORs) and the corresponding 95% confidence intervals (CIs) were used to measure the association of the selected SNPs with MDD in dominant and recessive models, respectively. Calculations were performed using SPSS 17.0 (SPSS Inc., Chicago, IL, USA). To adjust for multiple testing, the level of significance was corrected via Bonferroni correction. All tests were two-tails, and the significance level was set at 0.05.

## Additional Information

**How to cite this article**: Zhang, C. *et al*. Identification of IL6 as a susceptibility gene for major depressive disorder. *Sci. Rep.* **6**, 31264; doi: 10.1038/srep31264 (2016).

## Supplementary Material

Supplementary Information

## Figures and Tables

**Table 1:** Comparison of genotype and allele frequencies of *IL6* SNPs between MDD and control groups.

SNP	n	Genotype			*P*	OR (95% CI)	*P*	*P*^ c^	n	Allele		OR (95% CI)	*P*
rs1800797		AA	AG	GG						A	G		
Case	772	0	19 (2.5)	753 (97.5)	0.002	4.76 (1.61–14.07)^a^	0.002^a^		1544	19 (1.2)	1525 (98.8)	4.72 (1.60–13.89)	0.002
Control	759	0	4 (0.5)	755 (99.5)		NA^b^	NA^b^	0.94	1518	4 (0.3)	1514 (99.7)		
rs1800796		GG	GC	CC						G	C		
Case	772	56 (7.3)	346 (44.8)	370 (47.9)	0.48	1.06 (0.87–1.30)^a^	0.57^a^		1544	458 (29.7)	1086 (70.3)	1.01 (0.86–1.18)	0.93
Control	759	64 (8.4)	320 (42.2)	375 (49.4)		0.85 (0.59–1.23)^b^	0.39^b^	0.72	1518	448 (29.5)	1070 (70.5)		
rs1800795		CC	CG	GG						C	G		
Case	772	0	16 (2.1)	756 (97.9)	0.06	2.27 (0.93–5.56)^a^	0.09^a^		1544	16 (1.0)	1528 (99.0)	2.26 (0.93–5.51)	0.07
Control	759	0	7 (0.9)	752 (99.1)		NA^b^	NA^b^	0.90	1518	7 (0.5)	1511 (99.5)		
rs2069837		GG	GA	AA						G	A		
Case	772	22 (2.8)	269 (34.8)	481 (62.3)	0.70	1.06 (0.86–1.30)^a^	0.60^a^		1544	313 (20.3)	1231 (79.7)	1.03 (0.86–1.23)	0.76
Control	759	25 (3.3)	251 (33.1)	483 (63.6)		0.86 (0.48–1.54)^b^	0.66^b^	0.27	1518	301 (19.8)	1217 (80.2)		
rs1524107		CC	CT	TT						C	T		
Case	772	58 (7.5)	354 (45.9)	360 (46.6)	0.68	1.04 (0.85–1.27)^a^	0.72^a^		1544	470 (30.4)	1074 (69.6)	1.00 (0.86–1.17)	1.00
Control	759	64 (8.4)	334 (44.0)	361 (47.6)		0.88 (0.61–1.28)^b^	0.51^b^	0.28	1518	462 (30.4)	1056 (69.6)		

^a^*P* values in dominant model.

^b^*P* values in recessive model.

^c^*P* values for Hardy-Weinberg equilibrium in control group.
