# Supplementary material for: Identification of IL6 as a susceptibility gene for major depressive disorder
Source: Sci Rep. 2016 Aug 9;6:31264. doi: 10.1038/srep31264 (PMC4977523; doi:10.1038/srep31264)

**Identification of IL6 as a susceptibility gene for major depressive disorder**

Chen Zhang ^a^,*, Zhiguo Wu ^a^, Guoqing Zhao ^a^, Fan Wang ^a^, Yiru Fang ^a^,*

^a^ Division of Mood Disorders, Shanghai Mental Health Center, Shanghai Jiao Tong University School of Medicine, Shanghai, China

Running title: IL6 and MDD

*Corresponding author.

E-mail address: [zhangchen645@gmail.com](mailto:zhangchen645@gmail.com) (C. Zhang)c

E-mail address: [yirufang@aliyun.com](mailto:yirufang@aliyun.com) (Y. Fang)

Supplementary Table S1 Demographics of MDD cases and controls for expression analyses

|  | Cases | Controls | *P* |
| --- | --- | --- | --- |
| Number of subjects (n) | 50 | 50 |  |
| Age (years), mean (SD) | 29.2 (6.0) | 30.8 (6.1) | 0.19 |
| Gender, male n (%) | 17 (34.0) | 21 (42.0) | 0.54 |
| Smoking status, n (%) | 13 (26.0) | 15 (30.0) | 0.82 |
| Body mass index | 22.7 (1.4) | 23.3 (1.0) | 0.03 |
| Alcoholic abuse, n (%) | 0 (0) | 0 (0) |  |
| Duration of illness (month) a, mean (SD) | 2.9 (1.0) | N/A |  |
| HRSD-17 b, mean (SD) | 24.9 (2.2) | N/A |  |
| Number of episode, mean (SD) | 1.4 (0.4) | N/A |  |
| Family history of mood disorders, n (%) | 3 (6.0) | N/A |  |

Note: ^a^ Duration of illness prior to admission

^b^ HRSD-17 on admission

Supplementary Table S2 Results of the pairwise haplotype test between MDD and control groups

| Haplotype^a^ | Frequency (%) | |  |
| --- | --- | --- | --- |
| rs2069837- rs1524107 | cases | controls | *P* ^b^ |
| A-T | 69.6 | 69.6 | 1.00 |
| G-C | 20.3 | 19.8 | 0.76 |
| A-C | 10.2 | 10.6 | 0.69 |

^a^ Haplotypes with frequency ＜3% are ignored in analysis.

^b^ *P* values for single haplotype test, d.f.=1, not corrected for multiple test.

Supplementary Table S3 Information of selected SNPs genotyped in this study

| SNP | Chromosome | Allele | Position | SNP property |
| --- | --- | --- | --- | --- |
| rs1800797 | 7 | G/A | 22726602 | 5’-flanking |
| rs1800796 | 7 | G/C | 22726627 | 5’-flanking |
| rs1800795 | 7 | G/C | 22727026 | 5’-flanking |
| rs2069837 | 7 | A/G | 22728408 | Intron 2 |
| rs1524107 | 7 | C/T | 22728600 | Intron 2 |

Supplementary Figure S1. Linkage disequilibrium plots consisting of 5 SNPs at the *IL6* gene Pairwise linkage disequilibrium (LD) was computed for all possible combinations using the values of *r^2^*×100.


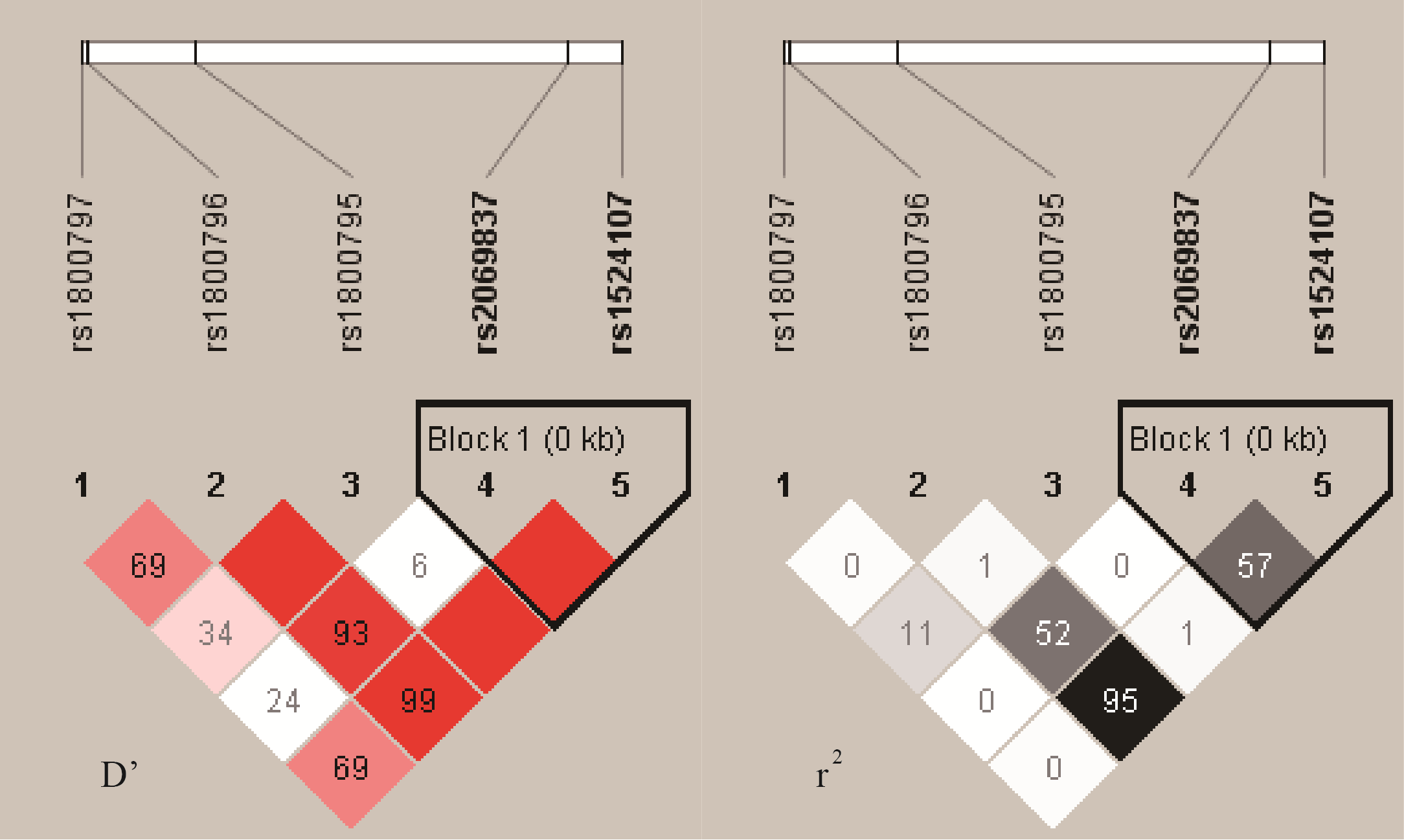

Supplement: Supplementary Information [file srep31264-s1.doc]
